# Plant-based dietary miRNAS: cross-border regulatory factors for regulating glycolipid metabolism and their nutritional intervention strategies

**DOI:** 10.3389/fnut.2025.1703178

**Published:** 2025-11-27

**Authors:** Yaping Liu, Xingyue Tao, Xiaoxiao Wang, Huilin Lv, Huomin Luo, Peifeng Li

**Affiliations:** 1 College of Food and Bioengineering, Zhengzhou University of Light Industry, Zhengzhou, Henan, China; 2 Institute of Life and Health, Zhengzhou University of Light Industry, Zhengzhou, Henan, China; 3 Henan Key Laboratory of Cold Chain Food Quality and Safety Control, Zhengzhou University of Light Industry, Zhengzhou, China; 4 Key Laboratory of Cold Chain Food Processing and Safety Control, Ministry of Education, Zhengzhou University of Light Industry, Zhengzhou, China

**Keywords:** plant miRNA, exosomes, cross-kingdom regulation, glucose and lipid metabolism, nutritional intervention

## Abstract

Disorders of glucose and lipid metabolism represent a major global health burden, driving the need for innovative nutritional interventions. Emerging evidence suggests that dietary microRNAs (miRNAs) from plants can act as cross-kingdom regulators. These molecules may survive digestion, enter the mammalian circulation, and modulate host gene expression to influence glycolipid homeostasis. This review comprehensively synthesizes current knowledge in this field, beginning with the dietary origins of plant miRNAs and evaluating their stability during food processing and gastrointestinal passage. We critically examine the mechanisms and ongoing debates regarding their absorption, transport, and biodistribution. The review further elucidates how specific plant miRNAs target key genes and signaling pathways involved in glucose and lipid metabolism, while also exploring their interplay with gut microbiota and metabolic inflammation. We summarize supporting evidence from *in vitro* and animal models, acknowledge translational challenges in human applications, and discuss persistent controversies regarding their bioavailability and biological relevance. Looking forward, we explore potential strategies to harness these molecules for nutrition therapy, including dietary source optimization, advanced processing techniques, engineered delivery systems, and precision nutrition approaches based on individual metabolic and miRNA profiles. Finally, we identify major knowledge gaps and future research priorities, such as clarifying uptake mechanisms, validating efficacy in well-controlled human trials, and establishing safety and regulatory frameworks. This review provides a foundational understanding and forward-looking perspective on plant-derived dietary miRNAs as novel factors in nutrition and metabolic disease management.

## 1 Introduction

Globally, disorders of glucose and lipid metabolism—such as obesity, type 2 diabetes, and non-alcoholic fatty liver disease—have emerged as major public health concerns. According to the 11th edition of the International Diabetes Federation (IDF) Atlas, the number of adults aged 20–79 years with diabetes reached 589 million in 2024, and this figure is projected to rise to 853 million by 2050. Concurrently, obesity, a central driver of these metabolic disorders, exhibits an equally alarming prevalence: in 2021, approximately 2.1 billion adults (≥25 years old) were overweight or obese, accounting for 45.1% of the global adult population. If current trends continue, this number is expected to surge to 3.8 billion by 2050, affecting nearly 60% of the world’s adult population (1). In light of this severe disease burden, exploring novel nutritional intervention strategies is of critical importance for the prevention and management of glucose and lipid metabolic disorders.

MicroRNAs (miRNAs) are key small non-coding RNAs that regulate gene expression at the post-transcriptional level. Highly conserved and endogenous, they have been primarily recognized for their roles in modulating gene expression within organisms (2–4). However, the recently proposed concept of “cross-kingdom regulation” has challenged this conventional understanding. Studies utilizing engineered delivery systems (e.g., lentiviral vectors) have demonstrated that plant miRNAs can regulate key target genes in mammals (5). For instance, *Lycium barbarum* Lb-miR166a inhibits the progression of triple-negative breast cancer by targeting STK39 and suppressing the MAPK14 pathway (6). Similarly, MIR2911 from honeysuckle can be absorbed through the diet, secreted within host small extracellular vesicles (sEVs), and directly act on gut bacteria to reduce Shigella abundance, thereby alleviating colitis symptoms. This positions MIR2911 as a novel therapeutic target for colitis treatment (7).

Nevertheless, the field remains contentious (8, 9). Skeptical studies argue that dietary miRNAs are prone to degradation during digestion, and their detected abundance in plasma is too low to reach biologically effective thresholds (10, 11). In addition, methodological challenges—such as potential contamination by plant RNA—may lead to false-positive conclusions (12). Despite these debates, advances in engineered delivery strategies are actively addressing absorption barriers. Thus, clarifying the feasibility, mechanisms, and limitations of plant miRNA-mediated cross-kingdom regulation is essential for developing RNA-based dietary interventions for metabolic diseases (13).

Although the mechanisms and biological activities of cross-kingdom regulation by plant-derived miRNAs remain debated, emerging evidence suggests that certain dietary plant miRNAs can cross species barriers, enter the mammalian circulatory system, and regulate key host genes and signaling pathways involved in glucose and lipid homeostasis (5, 14). For example, studies have shown that plant miRNAs can influence core metabolic processes such as insulin sensitivity, hepatic gluconeogenesis, adipocyte differentiation, and cholesterol metabolism (15). To systematically evaluate recent advances in this field and assess its translational potential for nutritional interventions in metabolic diseases, this review will focus on the following key aspects: (1) the primary dietary sources of plant-derived miRNAs, along with evidence and challenges regarding their stability in complex food matrices and gastrointestinal environments, as well as their absorption and transport mechanisms; (2) molecular targets and pathways through which representative plant miRNAs regulate glucose and lipid metabolism, and their potential interactions with metabolic inflammation and gut microbiota; (3) supporting evidence from *in vitro* and *in vivo* studies, and current challenges in human research (16); (4) the potential and specific strategies for developing innovative nutritional interventions based on plant-derived miRNAs, alongside associated scientific, technological, and regulatory challenges.

Based on the above key points, we first explore the dietary sources of plant-derived miRNAs and their stability and absorption mechanisms during digestion.

## 2 Dietary sources and cross-kingdom journey of plant-derived miRNAs: stability and absorption mechanisms

### 2.1 Major dietary sources

Plant-derived dietary miRNAs are ubiquitous components of the human daily diet. Owing to distinct gene expression profiles across plant-based foods, the types and abundance of miRNAs vary considerably among different sources. These miRNAs are ingested through the consumption of either raw or processed plant foods, thereby initiating their cross-kingdom regulatory journey (17). Among them, certain miRNAs are considered “star molecules” due to their high abundance, stability, and demonstrated biological functions in experimental models, making them a major focus of current research. Common dietary plant-derived miRNAs and their functions are summarized in Tables 1, 2.

**Table 1:** Plant-derived miRNAs with evidence of cross-kingdom regulation in mammalian models.

Dietary source	Core miRNA molecule(s)	Main function	References
Rice	miR168a	Targets LDLRAP1 to inhibit hepatic LDL clearance; alleviates colitis.	(5, 87)
Cereals	miR156a	Attenuates hepatic steatosis and obesity, improves insulin sensitivity.	(34)
Cruciferous vegetables	miR159	Targets TCF7 and inhibits the growth of breast cancer cells.	(32)
Ginger	miR7267-3p	Targets mPGES-1 to exert anti-inflammatory effects and alleviate colitis.	(88)
Corn	miR166a	Inhibits mammary cell proliferation and promotes apoptosis via targeting APLNR.	(19, 89)
Honeysuckle	miR2911	Targets influenza A virus PB2 and NS1 genes, and inhibits viral replication.	(90)

**Table 2:** Native regulatory functions of dietary plant-derived miRNAs in plant biology.

Dietary source	Core miRNA molecule(s)	Main function	References
Rice	miR162a	Enhances resistance to brown planthopper via α-linolenic acid metabolism.	(91)
Vegetables	miR159	Regulates plant fertility and defense responses to biotic stress.	(92)
Tomato	miR1918	Enhances susceptibility to *Phytophthora infestans* infection.	(93)
Corn	miR168a miR166a	Regulates internode elongation and stem growth.	(19)
Arabidopsis	miR395c, miR395e	Responds to abiotic stress, affecting seed germination and seedling survival.	(94)

### 2.2 Stability challenges

For plant-derived miRNAs to successfully reach target organs and exert regulatory functions, they must overcome a series of formidable biological barriers. The stability challenges they face throughout the dietary intake pathway represent a core issue in evaluating their biological activity. The first hurdle occurs during food processing from “farm to fork.” Common physical processes such as juicing and grinding disrupt plant cell structures, releasing miRNAs from their native protective environments and rendering them more susceptible to degradation. Thermal processing methods—including cooking, boiling, and pasteurization—are among the most critical factors affecting miRNA stability. Studies have shown that boiling significantly reduces the levels of certain miRNAs in soy milk (18). Nevertheless, some miRNA molecules exhibit remarkable thermostability. For example, MIR168a remains detectable in substantial quantities in boiled corn (19) suggesting that different miRNAs may possess distinct thermal resistance properties.

After surviving food processing, ingested miRNAs must next endure the harsh environment of the human digestive system. The strongly acidic conditions of the stomach (pH ~ 1.5–3.5) can catalyze the hydrolytic cleavage of nucleic acids, while the intestine contains abundant ribonucleases (RNases)—highly efficient enzymes that degrade RNA (20). Additionally, bile salts act as biological detergents that disrupt protective vesicular structures, thereby exposing encapsulated miRNAs to enzymatic degradation (21). Together, these extreme conditions create an environment dedicated to the breakdown of biological macromolecules, posing a significant threat to the stability and retention of plant-derived miRNAs.

Despite these formidable challenges, numerous studies have detected intact plant-derived miRNAs in the circulatory systems and tissues of animals, indicating the evolution of sophisticated self-protection mechanisms to resist degradation. The most widely recognized mechanism is the encapsulation of miRNAs within exosome-like nanoparticles (ELNs). Rather than existing in a “naked” state, these miRNAs are enveloped in vesicles composed of a lipid bilayer (22). This lipid membrane acts as a physical barrier, effectively shielding the miRNAs from RNases and acidic conditions. For instance, ginger-derived ELNs have been shown to protect MIR7267-3p during its passage through the gastrointestinal tract (23). Additionally, the characteristic 2′-O-methylation modification at the 3′ end of eukaryotic miRNAs helps prevent degradation by exonucleases, significantly enhancing their stability (24). Another proposed mechanism suggests that plant miRNAs may form complexes with RNA-binding proteins such as Argonaute (AGO), which could further contribute to their protection and reduce the rate of clearance (25).

In summary, the ultimate biological effects of plant miRNAs depend on a dynamic balance between stability and degradation. Through naturally evolved strategies—such as encapsulation in ELNs and chemical modifications—these miRNAs partially overcome the severe challenges posed by food processing and digestion, thereby enabling their potential for cross-kingdom regulation (26). However, factors including food matrices, processing methods, and inter-individual digestive variations can significantly influence this balance, which also contributes to the ongoing controversy and heterogeneity observed in research findings in this field.

### 2.3 Absorption and transport mechanisms

Whether plant miRNAs can be effectively absorbed by the mammalian body and transported to distant organs to perform regulatory functions is a cornerstone of the cross-kingdom regulation hypothesis—and remains a highly debated topic in current research (27). Existing evidence presents a complex and still unresolved picture, with both supporting and contradictory findings contributing to a landscape of significant scientific uncertainty.

Evidence supporting the cross-kingdom absorption of plant miRNAs continues to accumulate. The central hypothesis posits that plant miRNAs are not taken up via passive diffusion, but rather through active or specialized cellular mechanisms. A key breakthrough was the identification of the SIDT1 (SID-1 Transmembrane family member 1) protein-mediated absorption pathway. This protein, primarily expressed on the apical membrane of mammalian intestinal epithelial cells, has been shown to mediate the efficient internalization of exogenous RNA (28). Studies indicate that mammalian intestinal cells actively uptake plant-derived miRNAs via an SIDT1-dependent mechanism, representing a critical first step in their entry into the body.

**Figure 1:**
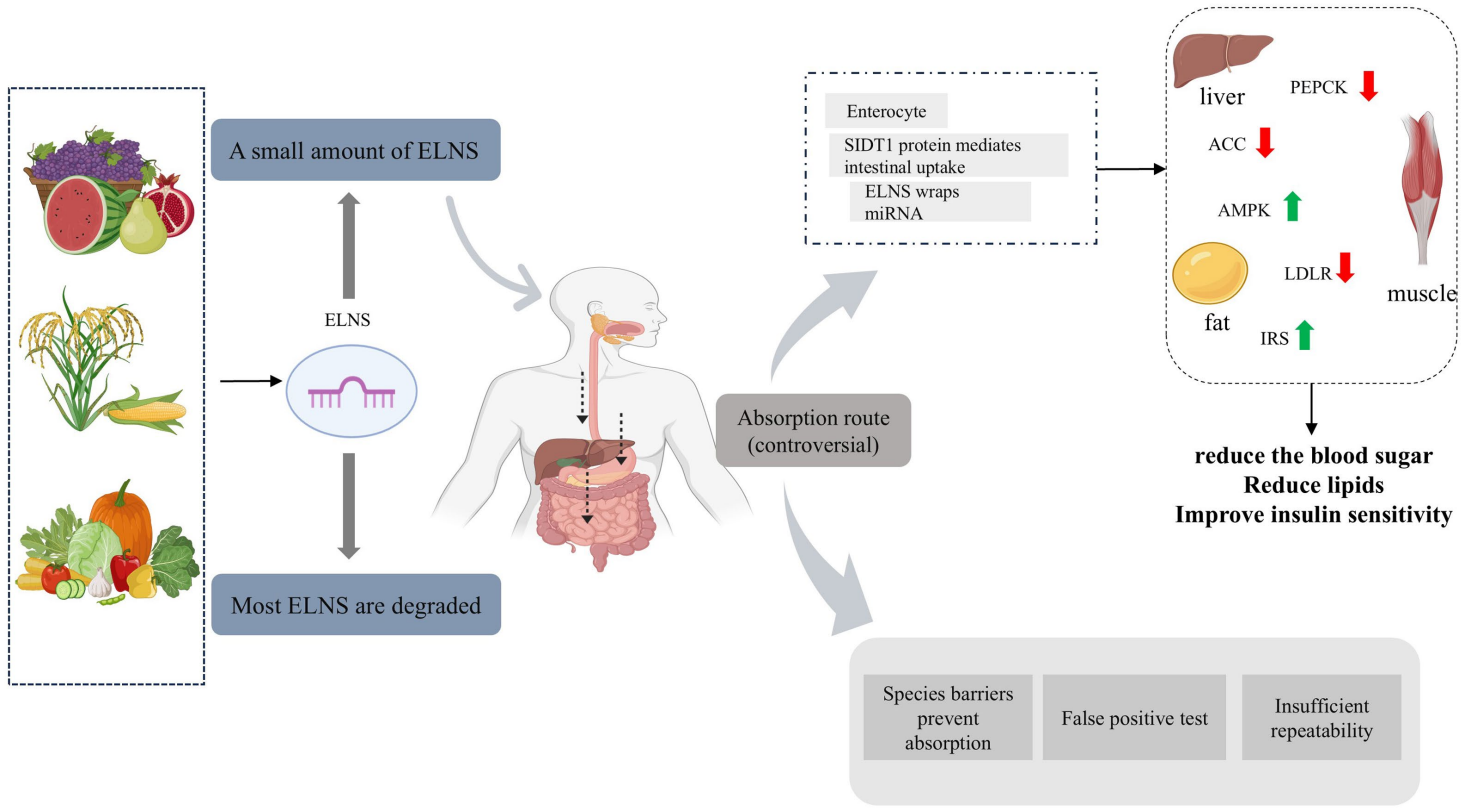
The cross-kingdom journey of dietary plant miRNAs: from absorption controversy to metabolic regulatory potential.

Once internalized, the release of these exogenous miRNAs into the circulatory system involves exosome-like nanoparticles (ELNs), which play a dual role: not only do they serve as protective carriers, but they may also function as transport vesicles (29). It is theorized that plant ELNs are taken up intact by intestinal cells via endocytosis. Subsequently, the miRNA cargo is released into the cytoplasm, where it may be reloaded into host-derived exosomes for systemic distribution via the lymphatic and circulatory systems (Figure 1) (30).

Ultimately, these circulating plant miRNAs can specifically enrich in key metabolic organs—such as the liver and adipose tissue—where they regulate host gene expression and physiological functions through canonical RNA interference mechanisms, including mRNA cleavage or translational repression. A notable example is MIR168a from rice, which has been shown to accumulate in the liver of mice and target the mRNA of low-density lipoprotein receptor adapter protein 1 (LDLRAP1), thereby modulating lipid metabolism. This study provides foundational evidence for a complete “ingestion–absorption–transport–function” pathway (31). After overcoming the absorption barrier, how plant miRNAs regulate the sugar and lipid metabolism of the host becomes a key issue. Below, we will delve into their molecular targets and pathways.

## 3 Key targets and pathways in the regulation of glucose and lipid metabolism

### 3.1 Regulation of glucose metabolism

The modulation of glucose metabolic homeostasis by plant-derived miRNAs involves a complex, multi-organ process (17). Current studies indicate that these miRNAs can simultaneously target multiple critical pathways—including insulin sensitivity, hepatic glucose output, and peripheral glucose uptake—suggesting their potential as multi-functional regulatory molecules. This provides a novel theoretical perspective on potentially improving insulin resistance and diabetes management.

Regarding the insulin signaling pathway, certain plant-derived miRNAs have been shown to directly target key nodes within the cascade, thereby enhancing insulin sensitivity. For instance, MIR159—derived from cruciferous vegetables—has been detected at considerable levels in human circulation, and its abundance is negatively correlated with prognosis in breast cancer patients (32). Further mechanistic studies revealed that MIR159 directly targets the TCF7 (Transcription Factor 7) gene in human breast cancer cells. As a key component of the Wnt signaling pathway, hyperactivation of TCF7-mediated Wnt signaling suppresses insulin signaling and promotes insulin resistance. By inhibiting TCF7, MIR159 indirectly stabilizes the degradation complex of *β*-catenin, attenuates Wnt-mediated interference with insulin signaling, and ultimately enhances cellular insulin sensitivity (33).

Additionally, MIR156a from rice has demonstrated notable anti-diabetic effects in a high-fat diet-induced obese mouse model. Its mechanism is associated with the modulation of expression and phosphorylation levels of insulin receptor substrates (IRS1/2) and the insulin receptor (INSR) in hepatic and adipose tissues, thereby potentiating insulin signal transduction and improving systemic glucose homeostasis (34).

In the regulation of hepatic glucose output, the liver—as the central organ of gluconeogenesis—represents an important target of plant-derived miRNAs. Abnormally activated hepatic gluconeogenesis is a major contributor to fasting hyperglycemia in type 2 diabetes (35). Studies have found that certain plant miRNAs can enter the systemic circulation, enrich in the liver (5). It is a subject of hypothesis that these miRNAs might target key rate-limiting enzymes in the gluconeogenic pathway. For example, it is plausible that plant miRNAs could affect the expression of PEPCK and G6Pase, possibly by mimicking or interfering with endogenous miRNA function. Theoretically, by suppressing these critical enzymes, plant-derived miRNAs could reduce endogenous glucose production, thereby lowering fasting blood glucose levels.

In promoting glucose uptake in peripheral tissues, skeletal muscle and adipose tissue serve as the primary sites of insulin-stimulated glucose disposal (36). Plant-derived miRNAs can also act on these tissues to enhance their glucose uptake capacity. The underlying mechanisms may involve: on one hand, enhancing the efficiency of insulin-stimulated translocation of glucose transporter 4 (GLUT4) to the cell membrane through the aforementioned insulin-sensitizing pathways; on the other hand, certain miRNAs may directly or indirectly modulate the activity of the AMPK signaling pathway (37). AMPK acts as a cellular energy sensor, and its activation promotes glucose uptake and utilization independently of insulin signaling. For example, MIR223 has been shown to activate AMPK in metabolic disorder models, thereby facilitating glucose clearance and utilization in skeletal muscle and adipose tissue, and ameliorating hyperglycemia (38).

### 3.2 Regulation of lipid metabolism

Plant-derived miRNAs also exhibit multi-target and network-like characteristics in the regulation of lipid metabolism, spanning core processes such as the maintenance of cholesterol homeostasis, fatty acid metabolic balance, and lipoprotein assembly and transport. These findings provide novel molecular insights into how dietary components can directly modulate host lipid metabolism and prevent related diseases (39).

In the regulation of cholesterol homeostasis, plant-derived miRNAs exert their modulatory effects through multi-target and multi-pathway mechanisms. Studies have shown that certain plant-derived miRNAs can influence cholesterol homeostasis. For example, the potential of dietary plant miRNAs to regulate cellular cholesterol efflux has been suggested (40). Furthermore, plant miRNAs can indirectly influence cholesterol metabolism by modulating the gut microbiota (7). For example, exogenous plant gma-miR-159a has demonstrated potential in regulating hepatic metabolic homeostasis. Functional screening has identified that gma-miR-159a effectively inhibits the activity of glycogen synthase kinase-3β (GSK-3β). In the progression of non-alcoholic fatty liver disease (NAFLD), overactivation of GSK-3β is a critical molecular node linking lipid metabolic disorders, insulin resistance, hepatic inflammation, and fibrosis. On one hand, GSK-3β suppresses insulin signaling and exacerbates hepatic lipid synthesis and deposition; on the other hand, it activates hepatic stellate cells, promoting their transformation into pro-fibrotic myofibroblasts and driving the release of pro-inflammatory factors. By targeting and inhibiting GSK-3β, gma-miR-159a not only improves hepatic insulin sensitivity and indirectly reduces *de novo* lipogenesis, but also directly suppresses the activation of hepatic stellate cells and liver inflammation. Thereby, it mitigates the progression of NAFLD to non-alcoholic steatohepatitis (NASH) and hepatic fibrosis through multiple mechanisms (41). These findings suggest that dietary plant miRNAs may serve as novel interventions for lipid metabolism-related liver diseases by targeting central signaling nodes.

In the context of fatty acid metabolism, it is noteworthy that plant exosomes, as natural carriers of miRNAs, may exhibit synergistic effects through their lipid components in combination with miRNAs. Recent studies have indicated that some plant exosome-like nanoparticles (PELNs) can modulate systemic lipid metabolism indirectly by regulating the intestinal microenvironment. For example, ginger-derived exosome-like nanoparticles (GDENs), upon oral administration, can withstand digestive conditions and be absorbed by intestinal cells. Research has demonstrated that GDENs upregulate the phosphorylation of AMP-activated protein kinase (AMPK), thereby coordinately regulating fatty acid metabolism: on one hand, they inhibit the activity of acetyl-CoA carboxylase (ACC) and fatty acid synthase (FAS), reducing *de novo* fatty acid synthesis; on the other hand, they promote the expression of carnitine palmitoyl transferase 1A (CPT1A), enhancing fatty acid *β*-oxidation. Consequently, GDENs significantly alleviate high-fat diet-induced hepatic steatosis and obesity (42).

In the regulation of lipoprotein metabolism, miRNAs can directly target apolipoproteins and key regulatory factors. Recent studies have shown that endogenous miRNAs, such as miR-369-3p, can regulate succinate metabolism and inflammasome activation by targeting the succinate receptor GPR91, thereby significantly influencing the formation of atherosclerotic plaques and inflammatory responses (43). Meanwhile, certain miRNAs can simultaneously inhibit the activity of sterol regulatory element-binding protein 1c (SREBP-1c) and enhance the expression of the low-density lipoprotein receptor (LDLR), thereby ameliorating atherogenic lipoprotein profiles through multi-target intervention (44).

Current research is shifting from single-molecule mechanisms to network-based regulatory analyses. By combining bioinformatic predictions with experimental validation, plant miRNAs have been suggested to integrate into host lipid metabolic networks via molecular mimicry (17). However, key questions remain regarding their biological effects at physiological concentrations, the interplay among different miRNAs, and species-specific differences. Further elucidation of these mechanisms will provide an important theoretical basis for developing plant miRNA-based strategies to intervene in lipid metabolic disorders.

### 3.3 Beyond direct regulation: interactions with metabolic inflammation and gut microbiota

The regulatory effects of plant-derived miRNAs on glucose and lipid metabolism are not limited to direct targeting of host genes. Growing evidence indicates that these miRNAs can also function as key signaling molecules that modulate crosstalk along the gut–liver and gut–adipose axes, influence the ecology of the gut microbiota and the composition of its metabolites, and subsequently regulate systemic metabolic inflammation. This provides a more comprehensive framework for understanding their role in ameliorating metabolic syndrome (45).

A complex bidirectional interaction exists between plant miRNAs and the gut microbiota. On one hand, the gut microbiota and microbiota-derived nucleases represent a major “barrier” that influences the stability and absorption efficiency of ingested plant miRNAs in the intestinal tract. On the other hand, and more intriguingly, plant miRNAs can actively shape the structure of the gut microbiota. Studies have shown that plant-derived exosome-like nanoparticles (ELNs) and their carried miRNAs can be taken up by specific gut bacteria, thereby regulating bacterial gene expression and growth (46). For example, garlic-derived exosome-like nanovesicles (GENs) are not only rich in bioactive components (such as lipids, proteins, and miRNAs) but can also carry specific miRNAs (e.g., Han-miR3630-5p) that target and inhibit TLR4 expression, modulating downstream inflammatory signaling pathways and significantly ameliorating DSS-induced colitis in mice (45).

Notably, this regulatory mechanism is particularly important in the gut environment associated with obesity and metabolic disorders. A high-fat diet can lead to gut microbiota dysbiosis, impaired barrier function, and a state of low-grade inflammation, thereby promoting metabolic endotoxemia and insulin resistance. Furthermore, GENs can modulate the composition of gut microorganisms, not only alleviating colonic inflammation but also potentially repairing obesity-associated damage to the intestinal mucosal barrier and reducing systemic inflammation (47). Short-chain fatty acids (SCFAs), such as butyrate, have been shown to enhance intestinal barrier function, suppress inflammation, and improve energy metabolism, which are closely linked to anti-obesity effects.

The liver and adipose tissue are primary sites of metabolic inflammation (48). Under conditions such as obesity, gut dysbiosis can compromise intestinal barrier integrity, enabling the translocation of pathogen-associated molecular patterns (PAMPs)—such as lipopolysaccharide (LPS)—into the portal circulation. This subsequently activates immune cells (e.g., macrophages) in the liver and adipose tissue, triggering chronic low-grade inflammation, also known as metabolic inflammation, which is a key contributor to insulin resistance (49). Through their modulatory effects on gut microbiota, plant-derived miRNAs may enhance intestinal barrier function, reduce LPS entry into the bloodstream, and mitigate inflammatory triggers at their source.

**Figure 2:**
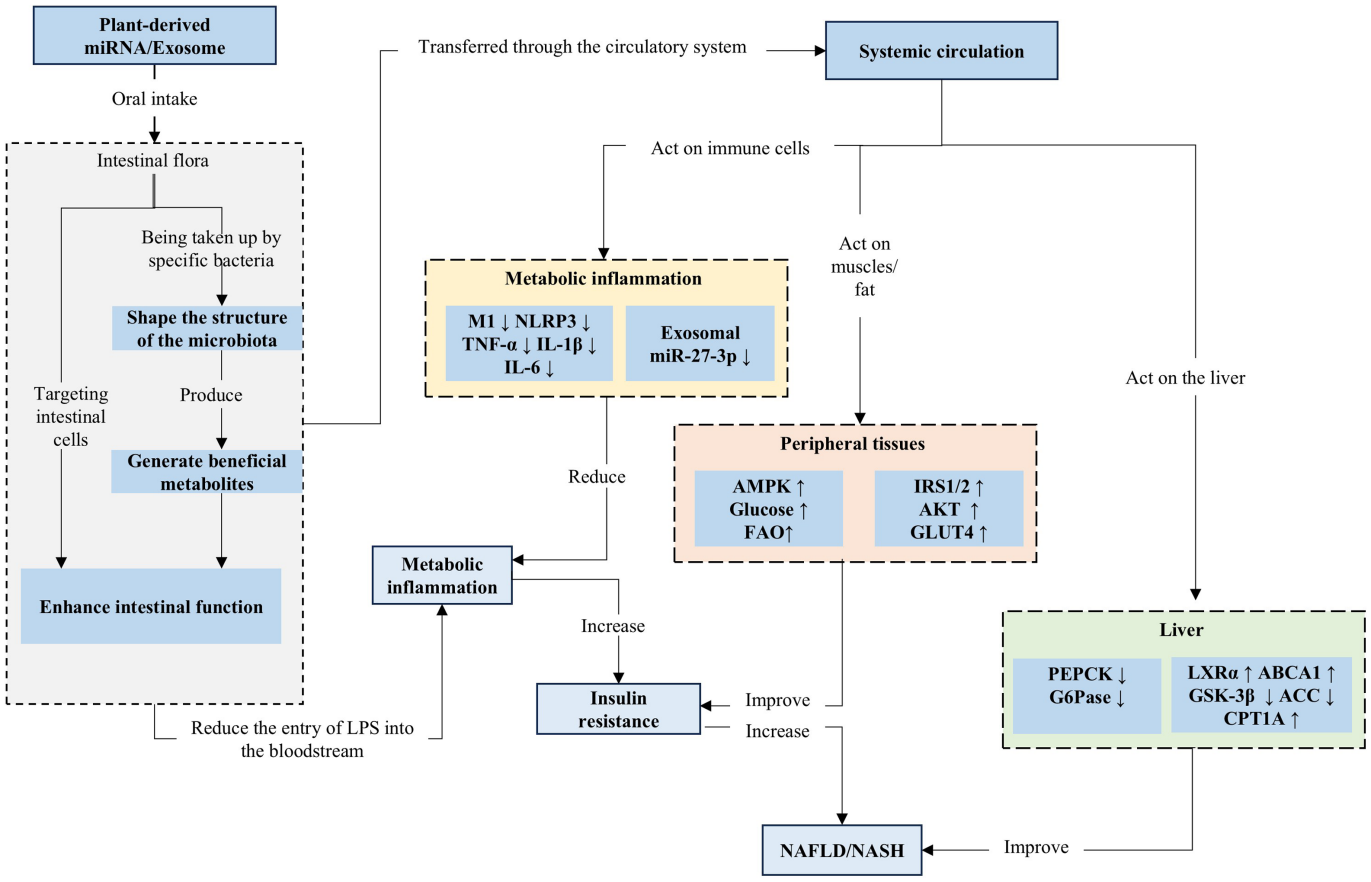
Mechanism of plant-derived miRNAs in the multi-organ regulation of glucose and lipid metabolism and metabolic inflammation.

Moreover, certain plant-derived miRNAs inherently possess the ability to suppress macrophage-driven inflammatory responses (50). In obesity and related metabolic disorders, the functional state of macrophages and the miRNAs carried by their exosomes also play critical roles. For instance, recent research has shown that under lipotoxic stress (e.g., LPS combined with palmitic acid), the expression of miR-27-3p is significantly elevated in exosomes derived from M1-polarized macrophages. This miRNA exacerbates insulin resistance by inhibiting its target gene Miro1 (mitochondrial GTPase 1), leading to impaired mitochondrial fission, defective mitophagy, and activation of the NLRP3 inflammasome (51). This mechanism is particularly relevant in high-fat diet-induced obesity and type 2 diabetes, suggesting that exosomal miRNAs serve not only as conveyors of inflammatory signals but also as key molecular links between metabolic dysregulation and organellar dysfunction. Thus, the potential crosstalk between plant-derived miRNAs and immune cell-derived miRNAs in regulating metabolic inflammation offers a novel perspective for understanding the diet–microbiota–host immune dialog (Figure 2). This mechanism requires experimental verification. Therefore, in the following steps, we will summarize the evidence from *in vitro*, animal model, and human studies to assess the biological activity of plant miRNAs.

## 4 Experimental evidence: from *in vitro* and animal models to human studies

### 4.1 Evidence from *in vitro* cell models: cornerstone of mechanistic exploration

*In vitro* cell models provide a primary platform for precisely deciphering the molecular mechanisms of plant-derived miRNAs in a controlled environment. Through gain-of-function (e.g., transfection with mimics) or loss-of-function (e.g., inhibitor treatment) experiments in specific cell lines, researchers can directly validate their targets and signaling pathways (52). For instance, to investigate the cross-kingdom potential of plant miRNAs in metabolic regulation, researchers often screen candidate molecules using bioinformatic analyses followed by functional validation *in vitro*. A recent study focused on plant miR6262 demonstrated that transfection of its mimic into free fatty acid-treated HepG2 hepatocytes (mimicking a steatotic environment) and human multipotent adipose-derived stem cells (hMADs) differentiated into brown adipocyte-like cells significantly reduced the expression of the predicted target gene RXRA (retinoid X receptor alpha). This further modulated the expression profiles of downstream genes involved in metabolism and thermogenesis, including PPARA, G6PC, and SREBF1 in hepatocytes, as well as CIDEA, CPT1M, and PLIN1 in adipocytes (53). Although the miR6262 mimic did not significantly reduce lipid accumulation in hepatocytes or directly promote adipocyte browning in this model, the results still suggest that plant miRNAs possess the potential to cross species boundaries and regulate human metabolic gene expression, providing key *in vitro* evidence for understanding the role of dietary plant RNAs in metabolic homeostasis.

In addition, some plant-derived miRNAs exhibit direct regulatory effects on adipogenesis. For example, plant miR167e-5p has been shown to significantly promote the differentiation and lipid accumulation of 3 T3-L1 preadipocytes. Mechanistic studies revealed that it directly targets the 3′ untranslated region of *β*-catenin and suppresses its expression, thereby relieving the inhibition of key adipogenic transcription factors PPARγ and C/EBPα. This in turn upregulates downstream genes such as fatty acid-binding protein 4 (FABP4) and enhances lipid synthesis. Notably, treatment with a miR167e-5p inhibitor reversed these pro-lipogenic effects and restored *β*-catenin expression, confirming the specificity of its action (54). This study not only reveals a novel mechanism by which plant miRNAs regulate host adipogenesis but also suggests that diet-derived RNA molecules may directly participate in the regulation of systemic lipid metabolic balance. Although these *in vitro* studies cannot fully replicate the complexity of the *in vivo* environment, they provide an essential first step for elucidating direct target relationships and functional pathways.

### 4.2 Evidence from animal models: validation of physiological relevance

Animal models serve as a critical experimental system for evaluating the overall physiological effects and therapeutic potential of plant-derived miRNAs and their carriers, such as exosome-like nanoparticles (ELNs). These studies provide *in vivo* evidence that these dietary components can be absorbed following oral administration and exert systemic interventions in the progression of metabolic and inflammatory diseases by modulating inter-organ crosstalk, immune-inflammatory responses, and gut microecology. Key findings and mechanistic insights from representative studies are summarized in Table 3, which systematically illustrates the interventional effects and molecular mechanisms of plant-derived miRNAs and ELNs across different disease models.

**Table 3:** Examples of the effects of plant-derived miRNAs in animal models of metabolic diseases.

Source and component	Model	Administration	Effects	Key mechanisms	References
Ginger (G-ELN)	T2DM mice	Oral gavage	↓Fasting glucose, ↑glucose tolerance, ↓β-cell damage	Activates PI3K/Akt; enhances insulin sensitivity	(95)
Plant Extract*	NAFLD mice	Oral gavage (in drinking water or by diet)	↓Body weight, ↓hepatic steatosis, ↑insulin sensitivity, ↑lipid profile	Modulates miR-122/34a; improves gut microbiota	(96)
Zanthoxylum (ZbELNs)	Colitis mice	Oral administration	↓Disease index, ↑colon length, ↓inflammation	Targeted delivery; anti-inflammatory; barrier protection	(97)
Plum (PM-EVLP)	Colitis mice	Oral gavage	↓Colitis symptoms, ↓pathology	Targets macrophages; inhibits NLRP3 inflammasome	(98)
Rice (osa-miR168a)	Colitis mice	Oral administration	↑Gut barrier, ↓pathology	Survives digestion; activates Nrf2; inhibits NF-κB; modulates microbiota	(99)

These studies demonstrate that oral or injection administration of specific plant miRNAs or their enriched extracts can reproduce phenotypes of improved glucose/lipid metabolism, anti-inflammation, and gut microbiota modulation in disease models, providing strong supporting evidence for their bioactivity.

### 4.3 Challenges in human studies: bridging the gap from animals to clinical applications

Although *in vitro* and animal studies have provided a solid theoretical and preclinical foundation for the cross-kingdom regulatory mechanisms of plant-derived miRNAs, their translation into human research and applications remains highly challenging. Currently, high-quality clinical studies directly confirming the biological effects of dietary plant miRNAs in humans are still scarce. Major obstacles include: unclear stability, absorption efficiency, and bioavailability of plant miRNAs in the human gastrointestinal environment; high inter-individual variability in pharmacokinetic profiles, likely influenced by dietary context, gut microbiota composition, and host genetic background; and a lack of standardized, scalable techniques for the extraction, delivery, and *in vivo* tracing of plant miRNAs, which also restricts the design and implementation of clinical translational research. Future studies should focus on developing more sensitive detection technologies to track the distribution and function of exogenous miRNAs in humans, and conduct rigorously designed randomized controlled trials to systematically evaluate their safety, tolerability, and efficacy in diverse populations with metabolic diseases, thereby truly bridging the gap between animal models and human applications. Although human research faces challenges, the application potential of plant miRNAs in nutritional intervention is increasingly evident. In the following, we will explore the transformation strategies, including optimization of dietary sources and precise nutrition.

## 5 Application potential: toward precision nutrition intervention strategies

### 5.1 Screening of dietary sources and agronomic optimization

Acquiring specific plant-derived miRNAs with high abundance and demonstrated bioactivity is a key prerequisite for subsequent applications. Conventional dietary screening approaches are no longer sufficient to meet this demand, thus necessitating systematic optimization using modern molecular agronomic strategies, as systematically summarized in Table 4.

**Table 4:** Strategies and key technologies for optimizing dietary sources.

Strategy level	Core objective	Key technologies	Representative cases	References
Resource Screening	Identify high-expression germplasm	CRISPR/Cas9, Northern blot	Rice miR5504 regulates plant height and increases yield	(100, 101)
Cultivation Control	Induce endogenous expression	Light/water/nutrient stress	UV-B irradiation induces miRNA expression in *Arabidopsis*	(102–104)
Molecular Breeding	Develop stable high-expression varieties	Transcriptome & ChIP-seq analysis	miR396b/GRF6 module enhances rice salt tolerance	(105)

Through the aforementioned strategies, it is anticipated that “functionally enhanced” crops can be developed in the future. For instance, resource screening can identify elite germplasm that naturally exhibits high expression of specific miRNAs (55, 56), Coupled with cultivation control techniques, this approach can further induce the accumulation of target miRNAs within plants. Ultimately, molecular breeding can be employed to develop stable, high-expression varieties. These crops are expected to not only demonstrate superior agronomic traits—such as stress resistance and high yield—but also to be specifically enriched with miRNAs capable of regulating human glucose and lipid metabolism, exerting anti-inflammatory effects, or providing antiviral functions. Thereby, they could serve as reliable and efficient raw material sources for developing next-generation functional foods or precision nutritional supplements based on dietary miRNAs (57).

### 5.2 Innovative processing and storage technologies for stabilizing bioactivity

The stability of plant miRNAs throughout the food processing chain is a critical bottleneck determining their ultimate bioefficacy. Overcoming this challenge requires moving beyond traditional processing methods and developing targeted protection strategies. Novel non-thermal processing technologies, which utilize physical fields such as high pressure and electric fields for sterilization, avoid high-temperature treatments. These methods offer the significant advantage of maximally preserving heat-sensitive components while causing minimal alterations to product color and flavor, resulting in exceptionally high protective efficacy for miRNA stability (58). Furthermore, intelligent storage systems mitigate all forms of chemical degradation by precisely controlling key parameters such as temperature, atmosphere, and humidity. Their effectiveness in preserving miRNA stability ranges from moderate to high, depending on the specific packaging materials and technologies employed (59).

While these emerging technologies hold great promise, their practical application within the food industry still faces significant challenges. Although non-thermal processing technologies can protect miRNAs, they involve high equipment costs, limited processing capacity, and their universal applicability across different food matrices requires further validation. Intelligent storage systems, on the other hand, heavily depend on packaging materials and initial product quality, with data on their long-term stability still lacking. Therefore, when selecting stabilization strategies, it is essential to comprehensively consider the characteristics of the target miRNA, product costs, and the market positioning of the final product, rather than solely pursuing technological novelty.

### 5.3 Developing advanced delivery systems to overcome absorption barriers

Low absorption rate is a common challenge faced by exogenous RNA-based therapeutics. Drawing inspiration from pharmaceutical delivery strategies, the development of efficient and targeted oral delivery systems is central to enhancing the bioavailability of plant-derived miRNAs (Table 5) (60).

**Table 5:** Delivery systems for plant miRNAs.

Type	Features	Advantages	Challenges	References
Synthetic Nanocarriers	Natural vesicles; co-delivery; antibody targeting.	Biocompatible; combotherapy; immunostimulatory.	Production scaling; batch variation; long-term safety.	(106)
Engineered Plant Exosomes	Drug-loadable exosomes; surface modifiable.	Stable; targetable.	Purification difficulty; lack of standardization.	(107)
Microbial Carriers	Microbial vesicles; bioactive cargo.	Immune/metabolic regulation; barrier penetration.	Functional variability; insufficient PK/PD data.	(108)

The delivery systems summarized in Table 5 each possess distinct advantages and limitations, yet all currently remain in the early stages of development. Synthetic nanocarriers face challenges in scalable production and unknown long-term safety; engineered plant exosomes present difficulties in extraction and purification, along with a lack of standardized protocols; microbial carriers exhibit high functional variability, and pharmacokinetic data in humans are virtually absent. Future research must focus on bridging these translational gaps, for instance, by developing low-cost extraction methods based on agricultural byproducts or establishing unified quality control standards, to advance these promising delivery systems from laboratory research to potential future applications.

### 5.4 Developing multi-omics-based precision nutrition strategies

Precision nutrition represents an emerging strategy to address individual variability in glucose and lipid metabolism disorders. Its core principle lies in moving beyond the traditional “one-size-fits-all” dietary intervention model toward targeted regulation of specific metabolic phenotypes (61, 62). Plant-derived miRNAs, as dietary components with gene regulatory functions, serve as ideal vectors for implementing precision nutrition. Multi-omics-based precision nutrition frameworks aim to systematically integrate multi-level biological data to enable end-to-end personalized management—from the design of miRNA-based dietary interventions to efficacy evaluation (63). This framework primarily consists of the following three key steps:

Step 1: Deep Phenotyping. Leveraging multi-omics technologies enables fine stratification of target populations beyond conventional clinical indicators such as body mass index (BMI), blood glucose, and lipid profiles (64). Metagenomics characterizes individual gut microbiota composition; transcriptomics analyzes immunometabolic gene expression profiles in peripheral blood or adipose tissue; and metabolomics delineates dynamic changes in small-molecule metabolites in plasma or urine (65). Integrating these data with bioinformatic predictions of plant miRNA targets allows for the subcategorization of individuals with glucose and lipid metabolism disorders into subtypes such as “Inflammation-Dominant,” “Hyperactive Lipogenesis,” “Bile Acid Dysregulation,” or “Impaired Gut Barrier.” This stratification provides a theoretical basis for precisely matching intervention strategies (66).

Step 2: Predictive Matching and Intervention Design. A multidimensional database integrating “host phenotype–gut microbiota composition–dietary miRNA response” is established to screen for the most effective plant-derived miRNA combinations and enrichment carriers for specific subtypes using artificial intelligence algorithms (67–69). For instance, individuals with an “Inflammation-Dominant” subtype may be matched with plant miRNAs enriched for targeting the NLRP3 inflammasome pathway (70),while those with a “Hyperactive Lipogenesis” subtype could be prioritized for miRNAs regulating the AMPK pathway (39, 71). Concurrently, selected miRNAs are delivered using food engineering technologies—such as enriched functional foods or engineered plant exosome-based vectors—to enhance their targeting specificity and stability.

Step 3: Dynamic Monitoring and Feedback Adjustment. Throughout the intervention process, high-sensitivity technologies such as high-throughput sequencing and PCR are employed to dynamically monitor circulating levels of plant-derived miRNAs, changes in host gene expression, and evolution of the gut microbiota structure. These data are integrated with multi-timepoint measurements of clinical biochemical parameters, forming a closed-loop optimization system of “intervention–monitoring–evaluation–adjustment” (72–75). This enables real-time refinement of the types, dosages, and combinations of plant miRNAs, thereby dynamically maximizing the intervention efficacy.

While this multi-omics framework is theoretically comprehensive, significant obstacles exist in its practical implementation. Firstly, the generation and analysis of multi-omics data involve substantial costs, requiring extensive computational resources and bioinformatics expertise (63). Secondly, translating massive multi-omics data into actionable, straightforward clinical decisions (such as “which foods to consume”) remains a common challenge in the field of precision nutrition (61). Additionally, the dynamic nature of an individual’s microbiome and metabolome raises questions about the reliability of classifications based on single time-point measurements, necessitating validation through long-term studies (64). Consequently, in the short term, this highly personalized strategy may only be applicable to specific high-risk populations or research settings.

Nevertheless, by systematically defining these challenges, this framework establishes a clear agenda for future research (61). It underscores the urgent need for more affordable technologies, robust bioinformatic pipelines, and longitudinal study designs. In this way, the framework serves not only as a promising end goal but also as an essential guide for the step-by-step innovation required to make precision nutrition a widespread reality.

### 5.5 Challenges and translational prospects

Despite the promising prospects, the translational applications in this field still face three major challenges (Table 6).

**Table 6:** Major translational challenges and future research directions.

Category	Specific challenges	Research directions
Scientific Mechanisms	Efficacy at physiological concentrations unclear; significant individual variation.	Develop highly sensitive assays (ddPCR, SCS); validate using human organoids/organs-on-chips.
Technical Bottlenecks	High production costs; insufficient product standardization and stability.	Utilize agricultural byproducts for extraction; establish standardized QC; improve delivery system stability.
Safety and Regulation	Limited long-term safety data; lack of regulatory guidelines.	Perform chronic toxicity studies; assess genomic safety; promote academia-regulator collaboration for guidance.

Plant-derived miRNAs, as an emerging class of bioactive dietary components, continue to face debates and challenges. Nevertheless, the convergence of engineered delivery technologies and precision nutrition strategies may offer feasible technological pathways to overcome obstacles such as low absorption efficiency and high inter-individual variability (76, 77). Future research must uphold rigorous scientific principles, combining in-depth investigation into molecular mechanisms with parallel advances in applied engineering. Strengthening interdisciplinary collaboration will be essential to ultimately translate this promising field from basic research to clinical validation and industrial application, thereby providing novel nutritional solutions for the global prevention and management of glucose and lipid metabolism disorders. Based on the above application strategies, we can draw the conclusion that plant-derived dietary miRNAs offer a novel approach for the management of metabolic diseases.

## 6 Conclusion

Plant-derived dietary miRNAs have been proposed as an unprecedented “diet-host” communication mechanism, potentially introducing a novel paradigm in nutritional science. This review systematically demonstrates that these small RNA molecules, originating from daily diets, exhibit the potential to cross species barriers, enter the mammalian circulatory system, and potentially host gene expression—despite ongoing debates regarding their gastrointestinal stability and absorption efficiency. This capability may be facilitated by their encapsulation in exosome-like nanoparticles, unique chemical modifications, and potentially active uptake pathways mediated by proteins such as SIDT1 (78–80). More importantly, we have summarized suggesting that these miRNAs may modulate glucose and lipid metabolic homeostasis at multiple organ levels by targeting key pathways—including insulin signaling, hepatic glucose output, fatty acid synthesis, and cholesterol metabolism—as well as through complex interactions with the gut microbiota and immune system (40, 81). These findings contribute to expanding expand our understanding of dietary bioactive components and provide insights into how food constituents can may directly participate in the epigenetic regulation of the host.

However, translating this promising concept into tangible applications faces multiple challenges. Key scientific issues that must be addressed include the ongoing debate over their functional significance at physiological concentrations, substantial inter-individual response variability, technical bottlenecks in large-scale production and standardized quality control, as well as a lack of long-term safety data. To bridge these gaps, future research should prioritize the following concrete directions: First, establishing standardized protocols for the extraction, quantification, and bioactivity assessment of plant miRNAs is paramount. This includes defining reference materials and method validation criteria to enable cross-laboratory comparisons and data reproducibility (55, 56). Second, initiating large-scale, multi-center collaborative studies is crucial to verify the cross-kingdom journey and efficacy of key plant miRNAs, moving beyond single-laboratory findings to robustly validate their physiological relevance (7). Finally, creating open-access databases that integrate miRNA sequences, dietary sources, host targets, and clinical phenotypes will facilitate data sharing, mining, and the development of predictive models, accelerating the field toward precision nutrition application (69). Overcoming these obstacles is beyond the scope of any single discipline and necessitates deep collaboration across molecular biology, food science, clinical medicine, data science, and regulatory affairs (82, 83).

Despite the challenges ahead, the future remains promising. Through agronomic optimization to develop functionally enhanced crops, innovative processing technologies and engineered delivery systems to improve bioavailability, and especially the construction of multi-omics-based precision nutrition frameworks, plant-derived miRNAs could contribute to transformation of next-generation nutritional interventions (84–86). Ultimately, these advances may enable a leap from population-based to individualized nutrition, offering novel strategies—rooted in natural dietary components—for the global prevention and management of glucose and lipid metabolism disorders.

## Glossary

ABCA1: ATP-binding cassette transporter A1
ACC: Acetyl-CoA carboxylase
AGO: Argonaute
AMPK: AMP-activated protein kinase
BMI: Body mass index
CPT1A: Carnitine palmitoyl transferase 1A
CRISPR/Cas9: Clustered regularly interspaced short palindromic repeats/CRISPR-associated protein 9
ELNs: Exosome-like nanoparticles
FABP4: Fatty acid-binding protein 4
FAS: Fatty acid synthase
G6Pase: Glucose-6-phosphatase
GDENs: Ginger-derived exosome-like nanoparticles
GENs: Garlic-derived exosome-like nanovesicles
GLUT4: Glucose transporter type 4
GSK-3β: Glycogen synthase kinase-3 beta
GPR91: Succinate receptor 1
hMADs: Human multipotent adipose-derived stem cells
IDF: International Diabetes Federation
INSR: Insulin receptor
IRS1/2: Insulin receptor substrate 1/2
LDL: Low-density lipoprotein
LDLR: Low-density lipoprotein receptor
LDLRAP1: Low-density lipoprotein receptor adapter protein 1
LPS: Lipopolysaccharide
LXRα: Liver X receptor alpha
MAP K14: Mitogen-activated protein kinase 14
miRNA: MicroRNA
NAFLD: Non-alcoholic fatty liver disease
NASH: Non-alcoholic steatohepatitis
NLRP3: NLR family pyrin domain containing 3
PAMPs: Pathogen-associated molecular patterns
PEPCK: Phosphoenolpyruvate carboxykinase
PELNs: Plant exosome-like nanoparticles
PPARγ: Peroxisome proliferator-activated receptor gamma
RNases: Ribonucleases
RXRA: Retinoid X receptor alpha
SCFAs: Short-chain fatty acids
sEVs: Small extracellular vesicles
SIDT1: SID-1 transmembrane family member 1
SREBP-1c: Sterol regulatory element-binding protein 1c
STK39: Serine/threonine kinase 39
TCF7: Transcription factor 7
TLR4: Toll-like receptor 4
Wnt: Wingless-related integration site
